# Time From Initial Community‐Based Dementia Consultation to Confirmed Diagnosis: A Retrospective Analysis

**DOI:** 10.1111/psyg.70148

**Published:** 2026-02-16

**Authors:** Akiko Yamazaki, Masahiro Mishina, Yuki Sakamoto, Satoshi Suda

**Affiliations:** ^1^ Dementia‐Related Disease Medical Center Nippon Medical School Musashi Kosugi Hospital Kawasaki Kanagawa Japan; ^2^ Department of Neurology Nippon Medical School Tokyo Japan; ^3^ Department of Neurology Tokyo Rosai Hospital Tokyo Japan

**Keywords:** Alzheimer disease, community‐based consultation, dementia, diagnostic delay, mild cognitive impairment

## Abstract

**Background:**

With the launch of anti‐amyloid beta antibody drugs, physicians face the need to quickly diagnose dementia. In prehospital consultation services in Japan, the duration from memory‐related consultations to confirmed diagnosis has not been sufficiently investigated. Therefore, this study investigated the duration from initial community‐based dementia consultation to confirmed diagnosis and the factors associated with diagnostic delays across dementia subtypes.

**Methods:**

This retrospective observational study included patients who consulted the Community Consultation Center for Citizens with Mild Cognitive Impairment and Dementia (CCCCMD) and subsequently received a confirmed dementia diagnosis at Nippon Medical School Musashi Kosugi Hospital between 2010 and 2024. Time to diagnosis was defined as the number of days from the initial CCCCMD consultation to diagnostic confirmation. We classified those who were diagnosed with dementia during outpatient visits amongst the consultees as Alzheimer disease (AD) or non‐Alzheimer disease (non‐AD). Diagnostic delay was further divided into pre‐hospital interval and in‐hospital phase. Group comparisons and multivariable Cox proportional hazards and logistic regression analyses were performed to identify factors associated with diagnostic delay.

**Results:**

A total of 739 patients were included: 504 with AD and 235 with non‐AD dementias. Time to diagnosis was shorter in the AD group than in the non‐AD group. This difference was primarily attributable to prolonged delays during the pre‐hospital interval amongst patients with non‐AD; however, delays during the in‐hospital diagnostic process were small and largely overlapping between the groups. Diagnosis of AD, older age, and the presence of a primary care physician were associated with a shorter time to diagnosis, whereas higher cognitive scores at the initial community consultation were associated with longer diagnostic delays.

**Conclusion:**

Diagnostic delay differed substantially by dementia subtype. These findings highlight the importance of community‐based consultation pathways as key targets for reducing diagnostic delays, particularly for patients with non‐AD dementias.

## 1 Introduction

The ageing population and the launch of anti‐amyloid beta antibody drugs have increased the importance of early detection and intervention of dementia [1, 2]. Early diagnosis of dementia and mild cognitive impairment (MCI) offers the opportunity to expand treatment options, including pharmacological and non‐pharmacological treatments and interventions to support daily living [2]. However, many patients with dementia in Japan tend to visit medical institutions only after their condition has progressed to a moderate or severe stage, creating a large gap between the ideal of early diagnosis and the actual clinical situation [3]. Particularly, the absence of a regular primary care physician has been identified as a factor contributing to delays in seeking medical consultation for dementia in Japan [4]. Additionally, the pattern of initial symptom presentation, the process by which family members notice these changes, and triggers for seeking consultation or medical care may vary depending on the diagnostic subtype of dementia [5, 6, 7, 8].

**FIGURE 1:**
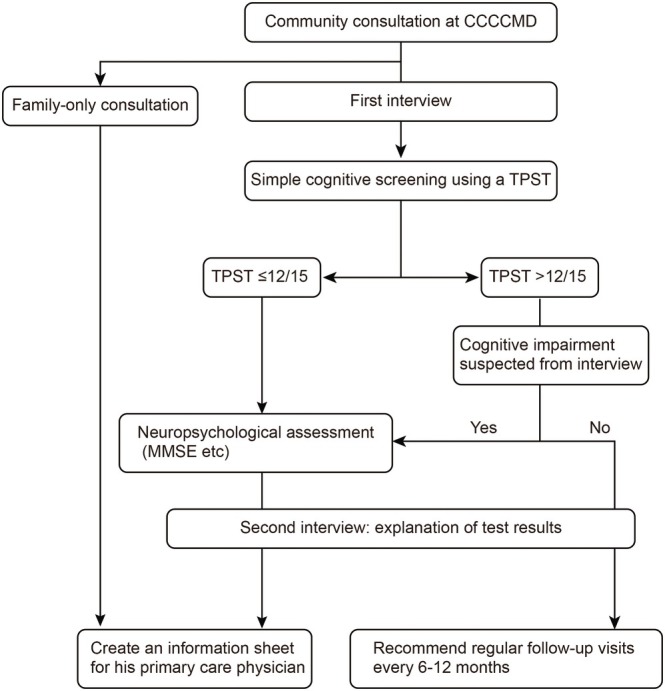
Consultation and referral process at the Community Consultation Center for Citizens with Mild Cognitive Impairment and Dementia (CCCCMD). At the initial consultation, visitors undergo a first interview conducted by trained clinical psychologists. When appropriate, simple cognitive screening is performed using the Touch Panel‐Type Dementia Assessment Scale (TPST). Individuals with a TPST score ≤ 12/15 or those in whom cognitive impairment is suspected based on the interview proceed to neuropsychological assessment, including the Mini‐Mental State Examination (MMSE). When necessary, an information sheet is prepared for the individual's family doctor. For individuals in whom cognitive impairment is not suspected, regular follow‐up consultations every 6–12 months are recommended. This consultation framework also includes family‐only consultations.

In order to detect cognitive impairment as early as possible, a project called the ‘Community Support Network for Citizens with Mild Cognitive Impairment and Dementia’ was launched in April 2017 [4]. The Community Consultation Center for Citizens with Mild Cognitive Impairment and Dementia (CCCCMD) was established as the core facility for the project. The CCCCMD operates as an out‐of‐hospital facility specialising in early detection and intervention of dementia, making it a unique initiative in Japan. The CCCCMD works in cooperation with local governments, such as Kawasaki City, and the community comprehensive support centres but also accepts consultations from outside the medical area. Free consultations are conducted by telephone or in person and are primarily provided by clinical psychologists. These consultations include interviews and brief cognitive assessments using the Touch Panel‐Type Dementia Assessment Scale (TPST) [4] and Mini‐Mental State Examination (MMSE). When deemed necessary, information letters addressed to primary care physicians or specialist medical institutions are prepared to encourage early medical consultation (Figure 1). The primary role of the CCCCMD is to determine whether referral to a medical institution is necessary and, when appropriate, to encourage early consultation. This structure lowers psychological barriers for individuals who are sensitive to their memory issues and hesitant to visit hospitals, making it easier for them to seek advice and visit centres.

The period from the first memory consultation to a definitive diagnosis has not been adequately investigated in dementia consultation services in Japan [4]. Therefore, the present study investigated individuals with memory complaints prior to hospital consultation and analysed the period from the initial consultation date at the CCCCMD to the date of definitive diagnosis at the Nippon Medical School Musashi Kosugi Hospital (NMSMKH), categorised according to the diagnostic classification. Diagnostic classification was divided into Alzheimer disease (AD) and non‐AD, and the study aimed to clarify differences in the number of days from initial consultation to confirmed diagnosis between these groups. Through this analysis, we sought to elucidate the characteristics of diagnostic delay according to the diagnostic classification and to obtain insights that may contribute to improving early diagnostic systems for dementia in community settings.

## 2 Methods

### 2.1 Study Design and Participants

This retrospective observational study was conducted at NMSMKH and the affiliated CCCCMD. Participants were patients who received a confirmed dementia diagnosis at the Department of Neurology at NMSMKH between 1 January 2010 and 31 December 2024. They had visited CCCCMD for an initial consultation.

### 2.2 Diagnosis and Classification

Board‐certified neurologists made the diagnoses based on standard clinical diagnostic criteria, neuropsychological assessments and neuroimaging findings, as documented in electronic medical records of NMSMKH. The first author reviewed the records and standardised the diagnostic labels to address inconsistencies. The dementia subtypes analysed included AD, Lewy body disease (LBD), vascular dementia (VaD) and primary age‐related tauopathy (PART). Patients with other diagnoses, such as MCI, subjective cognitive decline (SCD), treatable dementias (such as idiopathic normal pressure hydrocephalus), hypothyroidism and psychiatric disorders, were excluded from the analysis. The diagnosis of AD was primarily based on clinical criteria according to the National Institute on Ageing–Alzheimer's Association (NIA‐AA) guidelines [9], integrating findings from neuropsychological assessments and neuroimaging. In particular, neuroimaging evaluations included magnetic resonance imaging and cerebral perfusion imaging, such as single photon emission computed tomography (SPECT) with *N*‐isopropyl‐(^123^I)‐*p*‐iodoamphetamine (^123^I‐IMP), which were used as supportive tools for clinical diagnosis. In the interpretation of perfusion SPECT, distribution patterns considered characteristic of AD, including posterior cingulate cortex involvement as reported in previous studies [10], were referenced. Patients diagnosed as having LBD, VaD or PART were grouped as non‐AD for the purposes of analysis.

### 2.3 Data Collection

Data were collected from CCCCMD consultation records created in a database application, FileMaker and electronic medical records of NMSMKH. The electronic medical records of NMSMKH were imported into a database created in FileMaker using the data warehouse function. We linked the CCCCMD and NMSMKH databases using identification codes created using names and dates of birth, which allowed us to obtain information on whether patients visited NMSMKH and their NMSMKH identification number. The extracted variables included sex, age at the first visit, date of initial consultation, date of first hospital visit, date of confirmed diagnosis, diagnosis, educational history, MMSE scores at the initial CCCCMD consultation and at the time of diagnosis, living status (living alone or not) and primary care physician status. Medical records were reviewed if necessary, and patients with insufficient data to determine consultation, hospital visit or diagnosis dates were excluded. Using these data, we calculated the time to diagnosis (days = diagnosis date—consultation date) as the primary measure of diagnostic delay.

### 2.4 Outcome Measures

The primary outcome was the time in days from the initial consultation to the date of confirmed dementia diagnosis. Additionally, we defined three time intervals as outcome measures: (1) the time from the initial CCCCMD consultation to the first hospital visit, (2) time from the first hospital visit to confirmed dementia diagnosis and (3) time from the initial CCCCMD consultation to confirmed dementia diagnosis. Secondary outcomes included the associations between dementia subtype, age, sex and year of consultation with diagnostic delay. For analyses requiring dichotomisation of diagnostic delay, prolonged delay was defined using the median value of the outcome of interest. We also evaluated factors related to receiving an AD diagnosis at the initial visit.

### 2.5 Statistical Analysis

For each dementia subtype, descriptive statistics were calculated, including the median and interquartile range (IQR) as primary summary measures, as well as the mean and standard deviation. Group comparisons were conducted using the Kruskal–Wallis test, with a significance level set at *p* < 0.05. When significant differences were found, Steel–Dwass tests were performed for post hoc multiple comparisons. Time‐to‐event analyses were performed using Cox proportional hazards models, in which diagnostic delay was treated as a continuous variable. These models were used to evaluate the associations between diagnostic delay and dementia subtype, age, sex, educational history, MMSE scores, living status and primary care physician status. To evaluate temporal changes in factors associated with diagnostic delay, additional Cox proportional hazards analyses were performed with stratification by calendar period. The study period was divided into two phases, before 2017 and from 2017 onward, and separate models were constructed for each period. Moreover, logistic regression analyses were conducted as secondary analyses, in which diagnostic delay was dichotomised using the median value as the cutoff value. Independent variables included dementia subtype (AD versus [vs.] non‐AD), sex and age. Logistic regression analyses were also performed to identify factors associated with receiving an AD diagnosis at the initial CCCCMD consultation. All analyses were performed using the JMP (version 19.0.1; SAS Institute, Cary, North Carolina, USA).

### 2.6 Ethical Considerations

This study was approved by the Ethics Committee of the Nippon Medical School (approval number: M‐2025‐296) and was performed in accordance with the ethical standards laid down in the Declaration of Helsinki. All data used in the analysis were fully anonymised, ensuring that no personally identifiable information was included. Written informed consent was obtained from all participants at the time of their consultation at the community‐based dementia consultation centre, and an opt‐out approach was employed.

## 3 Results

### 3.1 Study Population

**FIGURE 2:**
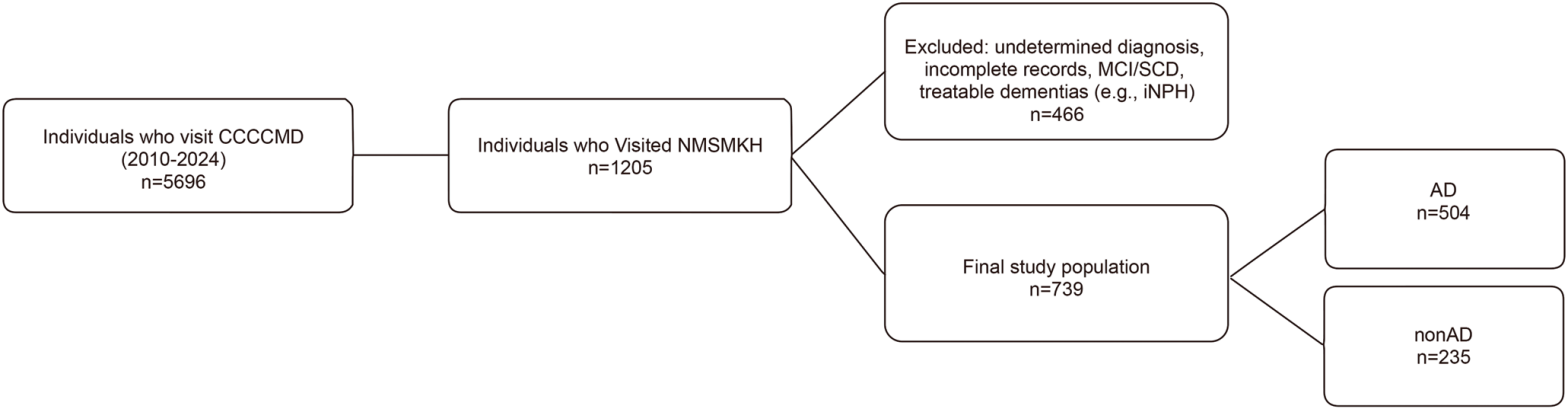
Study flow diagram from the initial Community Consultation Center for Citizens with Mild Cognitive Impairment and Dementia (CCCCMD) consultation to the final analysis. A total of 5696 individuals visited the CCCCMD between 2010 and 2024. Amongst them, 1205 subsequently visited Nippon Medical School Musashi Kosugi Hospital (NMSMKH). Individuals were excluded owing to no confirmed diagnosis, incomplete records, mild cognitive impairment (MCI) or subjective cognitive decline (SCD), or treatable dementias (e.g., idiopathic normal pressure hydrocephalus [iNPH]). The final study population consisted of 739 patients, including 504 with Alzheimer disease (AD) and 235 with non‐AD dementias. LBD, Lewy body disease; PART, primary age‐related tauopathy; VaD, vascular dementia.

According to the tabulation of the 2010–2020 national censuses of Kawasaki City, the population of the city increased from 1 425 512 in 2010 to 1 538 262 in 2020, whilst the proportion aged ≥ 65 years increased from 16.8% to 20.3% (national average in 2020: 28.6%). Against this demographic background of population growth and ageing [11], we analysed data from individuals who visited CCCCMD. A total of 739 patients were included in the final analysis (Figure 2).

### 3.2 Demographic Characteristics

Table 1 summarises the demographic and clinical characteristics of the study population. Of the 739 patients included in the final analysis, 504 were diagnosed with AD and 235 with non‐AD dementias. Overall, 65.8% of the participants were female, with a significantly higher proportion of women in the AD group than in the non‐AD group (69.3% vs. 58.2%, *p* = 0.0026).

**TABLE 1:** Demographic characteristics of patients.

Characteristic	AD (*n* = 504)	non‐AD (*n* = 235)	*p* ^a^
Female sex, *n* (%)	350 (69.4)	136 (57.9)	0.002
Age at first CCCCMD consultation, median (IQR), years	79 (74–83)	78 (74–82)	0.931
Time to diagnosis, median (IQR), days	112 (54–649)	239 (69–1475)	< 0.001
Time to first hospital visit, median (IQR), days	31 (7–385)	57 (15–836)	< 0.001
In‐hospital diagnostic time, median (IQR), days	49 (28–77)	56 (28–136)	0.022
Living alone, *n* (%)	92 (18.3)	38 (16.2)	0.496
Having a primary care physician, *n* (%)	405 (82.0)	207 (89.2)	0.012
Years of education, median (IQR)	12 (9–14)	12 (9–14)	—
MMSE at first CCCCMD consultation, median (IQR)	21 (17–24)	23 (18–25)	< 0.001
MMSE at diagnosis, median (IQR)	21 (16–23)	22 (18–24)	< 0.001

Abbreviations: AD, Alzheimer's disease; CCCCMD, Community Consultation Center for Citizens with Mild Cognitive Impairment and Dementia; IQR, interquartile range; MMSE, Mini‐Mental State Examination.

^a^ Wilcoxon test.

The median age at the first CCCCMD consultation was comparable between the AD and non‐AD groups (79 years [IQR 74–83] and 78 years [IQR 74–82], respectively, *p* = 0.931). However, the median time from the initial CCCCMD consultation to confirmed diagnosis was significantly shorter in the AD group than in the non‐AD group (112 days [IQR 54–649] vs. 239 days [IQR 69–1475], *p* < 0.001).

When the diagnostic process was divided into phases, patients with non‐AD dementias experienced significantly longer delays from the initial CCCCMD consultation to the first hospital visit (57 days [IQR 15–836] vs. 31 days [IQR 7–385], *p* < 0.001) and from the first hospital visit to diagnostic confirmation (56 days [IQR 28–136] vs. 49 days [IQR 28–77], *p* = 0.022).

There were no significant differences between the groups in the proportion of patients living alone or in years of education. Patients with non‐AD dementias were more likely to have a primary care physician than those with AD (89.0% vs. 82.2%, *p* = 0.0176). Median MMSE scores at the initial CCCCMD consultation and at diagnosis were significantly lower in the AD group than in the non‐AD group (both *p* < 0.001).

### 3.3 Diagnostic Delays by Category

**FIGURE 3:**
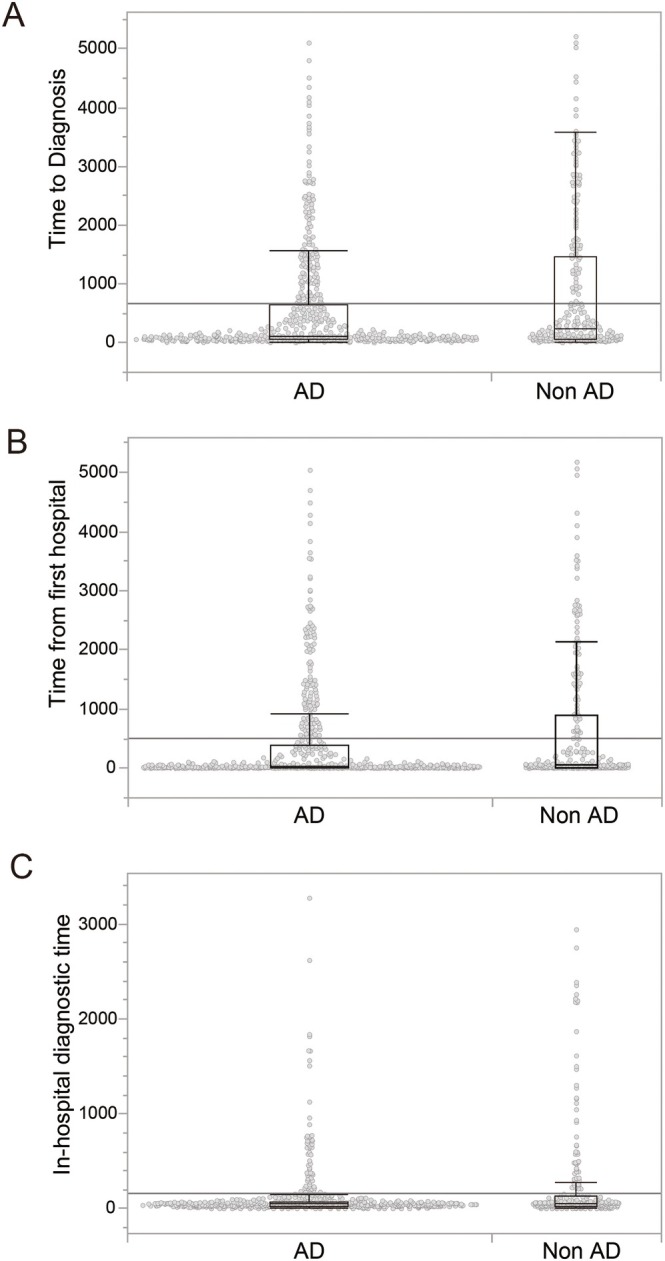
Comparison of time intervals across the diagnostic pathway between Alzheimer disease and non‐Alzheimer dementias. (A) Total time to diagnosis, defined as the interval from the initial community consultation to diagnostic confirmation. (B) Time from the initial community consultation to the first hospital visit. (C) In‐hospital diagnostic time, defined as the interval from the first hospital visit to diagnostic confirmation. Box plots represent the median and interquartile range (IQR), with whiskers indicating 1.5 × IQR. Individual data points are shown as jittered dots. AD, Alzheimer disease; CCCCMD, Community Consultation Center for Citizens with Mild Cognitive Impairment and Dementia.

Figure 3 shows boxplots comparing the time to diagnosis between patients with AD and those with non‐AD dementias across three diagnostic intervals. Across the entire diagnostic pathway from the initial CCCCMD consultation to confirmed diagnosis, patients in the non‐AD group showed a longer time to diagnosis and a markedly wider distribution than those in the AD group. The difference between the groups was statistically significant (Kruskal–Wallis test, *p* < 0.001). When the diagnostic process was divided into two phases, the group difference was most pronounced in the interval from the initial CCCCMD consultation to the first hospital visit. Patients with non‐AD exhibited greater variability and more extreme delays during this pre‐hospital phase, whereas those with AD tended to proceed to hospital consultation more promptly. In contrast, the time from the first hospital visit to confirmed diagnosis showed a smaller difference between the AD and non‐AD groups. Although some variability remained, the distributions largely overlapped, suggesting that in‐hospital diagnostic processes contributed less to the overall diagnostic delay than factors operating before hospital presentation.

Overall, the boxplots indicate that whilst AD cases were more tightly clustered with fewer extreme outliers, non‐AD cases demonstrated a broader distribution with higher maximum values, indicating that a subset of non‐AD patients experienced substantially prolonged diagnostic delays extending to several years.

### 3.4 Factors Associated With Diagnostic Delays

Cox proportional hazards models (Table 2) were used to examine factors associated with time to diagnosis across the entire diagnostic pathway, from the initial CCCCMD consultation to confirmed diagnosis. A diagnosis of AD was consistently associated with a shorter time to diagnosis than that of non‐AD dementias, indicating a higher hazard of reaching a confirmed diagnosis (hazard ratio [HR], 1.32; 95% confidence interval [CI], 1.12–1.57). Similar associations were observed across each diagnostic interval, including the time from the initial consultation to the first hospital visit and the time from the first hospital visit to diagnosis. Older age at the initial consultation was associated with a shorter time to diagnosis across all intervals. The presence of a primary care physician was also associated with a shorter time to diagnosis in the overall interval and in the interval from the initial consultation to the first hospital visit. In contrast, higher MMSE scores at the initial CCCCMD consultation were associated with a longer time to diagnosis in the overall interval and in the pre‐hospital phase. Sex was not significantly associated with overall diagnostic delay; however, female sex was associated with a shorter time to diagnosis in the pre‐hospital interval. Living status, MMSE score at diagnosis, and years of education showed no consistent associations with time to diagnosis across intervals.

**TABLE 2:** Cox proportional hazards models for diagnostic delay.

Variable	Overall diagnostic delay HR (95% CI)	Time to first hospital visit HR (95% CI)	In‐hospital diagnostic time HR (95% CI)
AD	1.32 (1.12–1.57)	1.24 (1.04–1.46)	1.37 (1.15–1.62)
Age at first CCCCMD consultation (years)	1.04 (1.02–1.05)	1.02 (1.01–1.04)	1.03 (1.01–1.04)
Female	0.86 (0.72–1.03)	0.82 (0.69–0.98)	1.01 (0.84–1.20)
Living alone	1.00 (0.81–1.24)	1.06 (0.86–1.32)	0.94 (0.76–1.17)
Having a primary care physician	0.60 (0.48–0.75)	0.59 (0.47–0.73)	0.80 (0.64–1.00)
MMSE at first CCCCMD consultation	0.98 (0.97–0.99)	0.98 (0.97–0.99)	0.98 (0.97–0.99)
MMSE at diagnosis	1.00 (0.99–1.01)	0.99 (0.98–1.00)	1.02 (1.00–1.03)
Years of education (years)	0.99 (0.96–1.02)	1.00 (0.97–1.03)	0.97 (0.94–1.00)

*Note:* HR > 1 indicates a shorter time to event.

Abbreviations: AD, Alzheimer's disease; CCCCMD, Community Consultation Center for Citizens with Mild Cognitive Impairment and Dementia; CI, confidence interval; HR, hazard ratio; MMSE, Mini‐Mental State Examination.

In complementary analyses using logistic regression analysis with a median‐based cutoff value for prolonged diagnostic delay (Table 3), AD was associated with a substantially lower likelihood of experiencing prolonged delay across all diagnostic intervals (overall: odds ratio [OR], 0.64; 95% CI, 0.45–0.91). This pattern was consistent for the pre‐hospital interval and the in‐hospital interval. Age ≥ 78 years was associated with a lower risk of prolonged delay in the overall interval, whereas it was associated with a higher risk of delay in the interval from the initial consultation to the first hospital visit. Female sex was associated with prolonged delay in the overall interval but showed a tendency towards shorter delay in the pre‐hospital phase. Higher educational attainment (≥ 12 years) was associated with a lower likelihood of prolonged diagnostic delay in the overall interval and in the pre‐hospital interval. An MMSE score ≥ 22 at the initial CCCCMD consultation was associated with a lower likelihood of prolonged diagnostic delay in the overall interval. An MMSE score ≥ 21 at diagnosis was associated with prolonged delay in the interval from the first hospital visit to confirmed diagnosis.

**TABLE 3:** Logistic regression analyses for prolonged diagnostic delay.

Variable	Overall delay ≥	Time to first hospital visit	In‐hospital diagnostic time
AD	0.64 (0.45–0.91)	0.69 (0.49–0.97)	0.65 (0.46–0.91)
Age ≥ 78 years	1.66 (1.20–2.31)	1.44 (1.04–2.00)	1.12 (0.82–1.54)
Female	1.53 (1.08–2.18)	1.60 (1.13–2.26)	0.90 (0.64–1.26)
Primary care physician	1.46 (0.94–2.24)	0.77 (0.51–1.18)	0.86 (0.57–1.30)
Education ≥ 12 years	0.35 (0.22–0.57)	0.33 (0.20–0.54)	0.83 (0.54–1.29)
Living alone	0.68 (0.48–0.96)	0.88 (0.63–1.23)	0.63 (0.45–0.88)
MMSE at CCCCMD ≥ 22	0.50 (0.29–0.87)	0.78 (0.46–1.33)	0.76 (0.45–1.28)
MMSE at diagnosis ≥ 21	1.18 (0.84–1.65)	0.89 (0.66–1.20)	1.36 (1.02–1.82)

*Note:* Results are presented as odds ratios (95% confidence intervals).

Abbreviations: AD, Alzheimer's disease; CCCCMD, Community Consultation Center for Citizens with Mild Cognitive Impairment and Dementia; MMSE, Mini‐Mental State Examination; Whole model test was *p* < 0.0001.

### 3.5 Temporal Changes in Factors Associated With Time to Diagnosis

The results of Cox proportional hazards models stratified by period (before and after 2017) are shown in Table 4. Across both periods, AD was consistently associated with a shorter time to diagnosis in all diagnostic intervals, with stronger associations observed after 2017. Older age was associated with a shorter time to diagnosis across all intervals before 2017; however, after 2017, age showed no clear association with time to diagnosis in the overall and pre‐hospital intervals, whilst an association persisted in the in‐hospital interval.

**TABLE 4:** Cox proportional hazards models stratified by period.

Variable	Overall diagnostic delay		Time to first hospital visit		In‐hospital diagnostic time	
	Before 2017	After 2017	Before 2017	After 2017	Before 2017	After 2017
	HR (95% CI)	HR (95% CI)	HR (95% CI)	HR (95% CI)	HR (95% CI)	HR (95% CI)
AD	1.58 (1.18–2.10)	1.60 (1.27–2.01)	1.40 (1.05–1.85)	1.45 (1.16–1.81)	1.44 (1.07–1.93)	1.60 (1.27–2.01)
Age at first CCCCMD consultation (years)	1.04 (1.01–1.06)	1.00 (0.98–1.01)	1.02 (1.00–1.04)	1.00 (0.99–1.01)	1.04 (1.01–1.06)	1.00 (0.98–1.01)
Female	1.08 (0.82–1.43)	0.80 (0.61–1.00)	1.19 (0.90–1.56)	0.84 (0.64–1.11)	0.92 (0.70–1.22)	0.67 (0.53–0.85)
Living alone	0.90 (0.64–1.27)	1.16 (0.88–1.53)	1.03 (0.74–1.44)	1.09 (0.82–1.44)	0.82 (0.58–1.14)	1.16 (0.88–1.53)
Having a primary care physician	0.55 (0.39–0.79)	0.61 (0.45–0.81)	0.59 (0.41–0.84)	0.61 (0.45–0.81)	0.57 (0.40–0.82)	0.89 (0.66–1.21)
MMSE score at first CCCCMD consultation	0.99 (0.97–1.00)	0.98 (0.96–1.00)	0.99 (0.98–1.01)	0.98 (0.96–1.00)	0.99 (0.97–1.01)	0.97 (0.95–0.99)
MMSE score at diagnosis	1.03 (1.01–1.05)	1.01 (0.99–1.02)	0.98 (0.96–0.99)	1.01 (0.99–1.02)	0.99 (0.97–1.01)	1.01 (0.99–1.02)
Years of education	0.99 (0.94–1.04)	0.95 (0.91–0.98)	1.00 (0.96–1.05)	0.95 (0.91–0.98)	1.00 (0.96–1.05)	0.95 (0.91–0.98)

*Note:* HR > 1 indicates a shorter time to event.

Abbreviations: AD, Alzheimer's disease; CCCCMD, Community Consultation Center for Citizens with Mild Cognitive Impairment and Dementia; CI, confidence interval; HR, hazard ratio; MMSE, Mini‐Mental State Examination.

After 2017, female sex was associated with a shorter time to diagnosis in the overall and pre‐hospital intervals, whereas no clear association was observed before 2017. Living alone showed no consistent association with time to diagnosis in either period. Having a primary care physician was associated with a shorter time to diagnosis in the overall and pre‐hospital intervals before 2017; after 2017, this association persisted only in the pre‐hospital interval.

Years of education were not associated with time to diagnosis before 2017; however, after 2017, a higher number of years of education was associated with a longer time to diagnosis in the overall and pre‐hospital intervals.

### 3.6 Factors Associated With AD Diagnosis at Initial Consultation

Table 5 shows the results of a logistic regression analysis identifying the factors associated with receiving an AD diagnosis at the time of the initial community‐based consultation. Female sex was significantly associated with a higher likelihood of an AD diagnosis (adjusted OR, 1.55; 95% CI, 1.09–2.21). Additionally, a higher cognitive score at the community consultation (MMSE score, ≥ 22) was independently associated with an AD diagnosis (adjusted OR, 1.85; 95% CI, 1.03–3.29). In contrast, age ≥ 78 years, living alone, educational attainment, the presence of a primary care physician, and MMSE score at diagnosis were not significantly associated with an AD diagnosis at initial consultation.

**TABLE 5:** Logistic regression analysis for factors associated with an AD diagnosis at initial consultation.

Variable	Adjusted OR (95% CI)
Female sex	1.55 (1.09–2.21)
Age ≥ 78 years	1.00 (0.71–1.41)
Living alone	1.02 (0.65–1.61)
Having a primary care physician	0.59 (0.35–0.99)
Education ≥ 12 years	1.28 (0.90–1.83)
MMSE at CCCCMD ≥ 22	1.81 (1.01–3.24)
MMSE at diagnosis ≥ 21	1.01 (0.55–1.82)

*Note:* Whole model test was *p* < 0.0001.

Abbreviations: AD, Alzheimer's disease; CCCCMD, Community Consultation Center for Citizens with Mild Cognitive Impairment and Dementia; CI, confidence interval; MMSE, Mini‐Mental State Examination; OR, odds ratio.

## 4 Discussion

This study examined the duration from the initial consultation at CCCCMD to the confirmation of a dementia diagnosis at NMSMKH in 739 patients and comprehensively analysed factors associated with diagnostic delay. The CCCCMD functions as a community‐based gateway where not only individuals themselves but also family members and local residents can seek consultation regarding memory problems and cognitive decline prior to visiting a hospital. A distinctive feature of this study is that it evaluated the entire diagnostic pathway, including the pre‐hospital phase, and examined the phases most prone to delay and their associated factors from multiple perspectives.

The main findings of this study indicate that patterns of diagnostic delay differ by dementia subtype. In particular, patients with non‐AD dementias experienced significantly longer times to diagnosis than those with AD, and this difference arose primarily during the pre‐hospital phase from the initial CCCCMD consultation to the first hospital visit. In contrast, delays during the in‐hospital diagnostic process from the first hospital visit to diagnostic confirmation were small in both groups, with substantial overlap in their distributions. These findings suggest that diagnostic delay may be driven more by factors operating in the pre‐hospital phase than by the in‐hospital diagnostic process itself.

The importance of the pre‐hospital phase was further supported by boxplot analyses. Amongst patients with non‐AD dementias, variability during this phase was particularly large, with some cases showing marked delays extending over several years, whereas distributions were more tightly clustered amongst patients with AD. Non‐AD dementias often present with heterogeneous and atypical clinical symptoms, which may complicate early symptom recognition, assessment of urgency, and referral decisions, thereby contributing to prolonged pre‐hospital delays. In contrast, AD more frequently presents with typical clinical features, which may facilitate earlier progression to hospital‐based evaluation.

Consistent with these observations, multivariable Cox proportional hazards analyses showed that an AD diagnosis was consistently associated with a shorter time to diagnosis even after adjustment for age, sex, cognitive function and social background. This finding suggests that AD may occupy an advantageous structural position within the overall diagnostic pathway. Moreover, the presence of a primary care physician was associated with a shorter time to diagnosis, particularly during the pre‐hospital phase, indicating that primary care may play an important role in influencing diagnostic trajectories.

Conversely, higher MMSE scores at the initial CCCCMD consultation were associated with longer times to diagnosis. This result suggests that in patients with milder cognitive impairment, observation or deferred decision making may be more likely to be selected or that atypical symptom profiles may make it difficult to establish an early diagnostic hypothesis, resulting in prolonged time to diagnostic confirmation. Similar tendencies were observed in logistic regression analyses using a median‐based definition of diagnostic delay, indicating that the complexity of diagnostic pathways in patients with milder symptoms may underlie this association.

Regarding sex differences, no consistent association with overall diagnostic delay was observed; however, during the pre‐hospital phase, women tended to reach hospital consultation more rapidly than men. Furthermore, analyses stratified by period demonstrated that after 2017, female sex was associated with shorter time to diagnosis in the overall and pre‐hospital intervals, suggesting that sex‐related differences in help‐seeking or consultation behaviour may have changed over time. Previous studies have reported that diagnoses of non‐AD dementias tend to be delayed [12], although most of them originated from outside Japan, and studies clearly demonstrating this pattern in Japanese populations remain limited.

Years of education showed no consistent association with diagnostic delay in the overall analysis; however, after 2017, longer educational attainment was associated with longer time to diagnosis. Although individuals with higher education might be expected to seek medical attention earlier, these findings suggest that factors such as differences in symptom interpretation, expectations regarding diagnosis or referral, preferences for observation or diversification of consultation pathways may have become more prominent in recent years than in previous years. Further investigation is required to clarify these mechanisms.

In analyses examining factors associated with receiving an AD diagnosis at the initial CCCCMD consultation, female sex and higher MMSE scores at the initial consultation were independently associated with an AD diagnosis. Compared with men, women represent a population with higher ageing rates and higher incidences of AD, and a higher prevalence of AD amongst women has been reported [13]. This association should be regarded as exploratory, given the observational design and limited covariate adjustment. Moreover, these findings may reflect the possibility that women recognise memory impairment earlier and that family members may be more likely to notice cognitive changes in women than in men [9]. Studies of health‐seeking behaviour further suggest that when memory problems are recognised, family members or surrounding individuals often encourage consultation [14]. Given that CCCCMD accepts consultations not only from individuals but also from family members and community residents, these social dynamics may contribute to the higher likelihood of an AD diagnosis amongst women than amongst men at the initial consultation.

## 5 Limitations

Some limitations of this study should be acknowledged. This was a single‐centre, retrospective observational study, and although it suggests factors associated with diagnostic delay, it does not establish causal relationships. Accordingly, the findings should be interpreted with a focus on associations rather than causation. Diagnoses were based on clinical assessment, and systematic biomarker evaluation was not performed in all cases; therefore, misclassification, particularly amongst non‐AD dementias, may have influenced the results. The study population was also a highly selected group limited to patients who were referred from CCCCMD to a specific medical institution and subsequently received a diagnosis, resulting in multiple sources of selection bias and limiting generalisability. In the general population based on the 2020 national census, the proportion of university graduates was 11.9%, whereas 18.1% of the study population had completed university education, indicating a highly educated cohort [11]. Additionally, 65.8% of participants were women, and the cohort was derived from a specific geographic region. Therefore, educational background, sex distribution and regional characteristics may have influenced the findings, and caution is warranted when applying these results to other populations or settings.

As a future research direction, it will be important to systematically categorise consultation content at CCCCMD, including chief complaints and consultation contexts, and examine their associations with diagnostic outcomes and help‐seeking behaviour. In particular, whilst the present study focused on individuals who ultimately visited a medical institution and received a diagnosis, future analyses incorporating individuals who consulted CCCCMD but did not proceed to hospital visits may provide a more comprehensive understanding of decision‐making processes and the structure of diagnostic delay during the pre‐hospital phase.

## 6 Conclusion

Diagnostic delays differed substantially by dementia subtype, with non‐AD dementias showing longer delays than AD, primarily during the pre‐hospital phase from initial community consultation to first hospital visit. These findings highlight community‐based consultation pathways such as the CCCCMD as important targets for strategies aimed at reducing diagnostic delays, particularly for non‐AD dementias.

## Disclosure

Dr. Mishina, Masahiro, a co‐author of this article, is an Editorial Board member of Psychogeriatrics. The authors declare no conflict of interests for this article.

## Ethics Statement

This study was approved by the Ethics Committee of the Nippon Medical School (approval number: M‐2025‐296).

## Consent

Written informed consent was obtained from all participants at the time of consultation, and an opt‐out approach was used in this retrospective study.

## Conflicts of Interest

The authors declare no conflicts of interest.

## Data Availability

The data that support the findings of this study are available on request from the corresponding author. The data are not publicly available due to privacy or ethical restrictions.
